# Synthesis and biological activities of the respiratory chain inhibitor aurachin D and new ring versus chain analogues

**DOI:** 10.3762/bjoc.9.176

**Published:** 2013-07-31

**Authors:** Xu-Wen Li, Jennifer Herrmann, Yi Zang, Philippe Grellier, Soizic Prado, Rolf Müller, Bastien Nay

**Affiliations:** 1 Muséum National d’Histoire Naturelle, Unité Molécules de Communication et Adaptation des Micro-organismes (UMR 7245 CNRS-MNHN), 57 rue Cuvier (CP 54), 75005 Paris, France; 2 Department of Microbial Natural Products, Helmholtz-Institute for Pharmaceutical Research Saarland (HIPS), Helmholtz Centre for Infection Research (HZI) and Pharmaceutical Biotechnology, Saarland University, Campus C2 3, 66123 Saarbrücken, Germany

**Keywords:** antiplasmodial activities, Conrad–Limpach reaction, mitochondrial membrane potential, natural products, quinolones, total synthesis

## Abstract

Aurachins are myxobacterial 3-farnesyl-4(1*H*)-quinolone derived compounds initially described as respiratory chain inhibitors, more specifically as inhibitors of various cytochrome complexes. They are also known as potent antibiotic compounds. We describe herein the first synthesis of aurachin D through a key Conrad–Limpach reaction. The same strategy was used to reach some ring as opposed to chain analogues, allowing for the description of structure–activity relationships. Biological screening of the analogues showed antiparasitic, cytotoxic, antibacterial and antifungal activities, and depletion of the mitochondrial membrane potential. The strongest activity was found on *Plasmodium falciparum* with a selectivity index of 345, compared to Vero cells, for the natural product and its geranyl analogue. The loss of mitochondrial membrane potential induced by aurachins in human U-2 OS osteosarcoma cells was studied, showing the best activity for aurachin D and a naphthalene analogue, yet without totally explaining the observed cytotoxic activity of the compounds. Finally, a synthetic entry is given to the complete carboheterocyclic core of aurachin H through the N-oxidation/epoxidation of aurachin D and a shorter chain analogue, followed by subsequent biomimetic cyclization.

## Introduction

**Figure 1:**
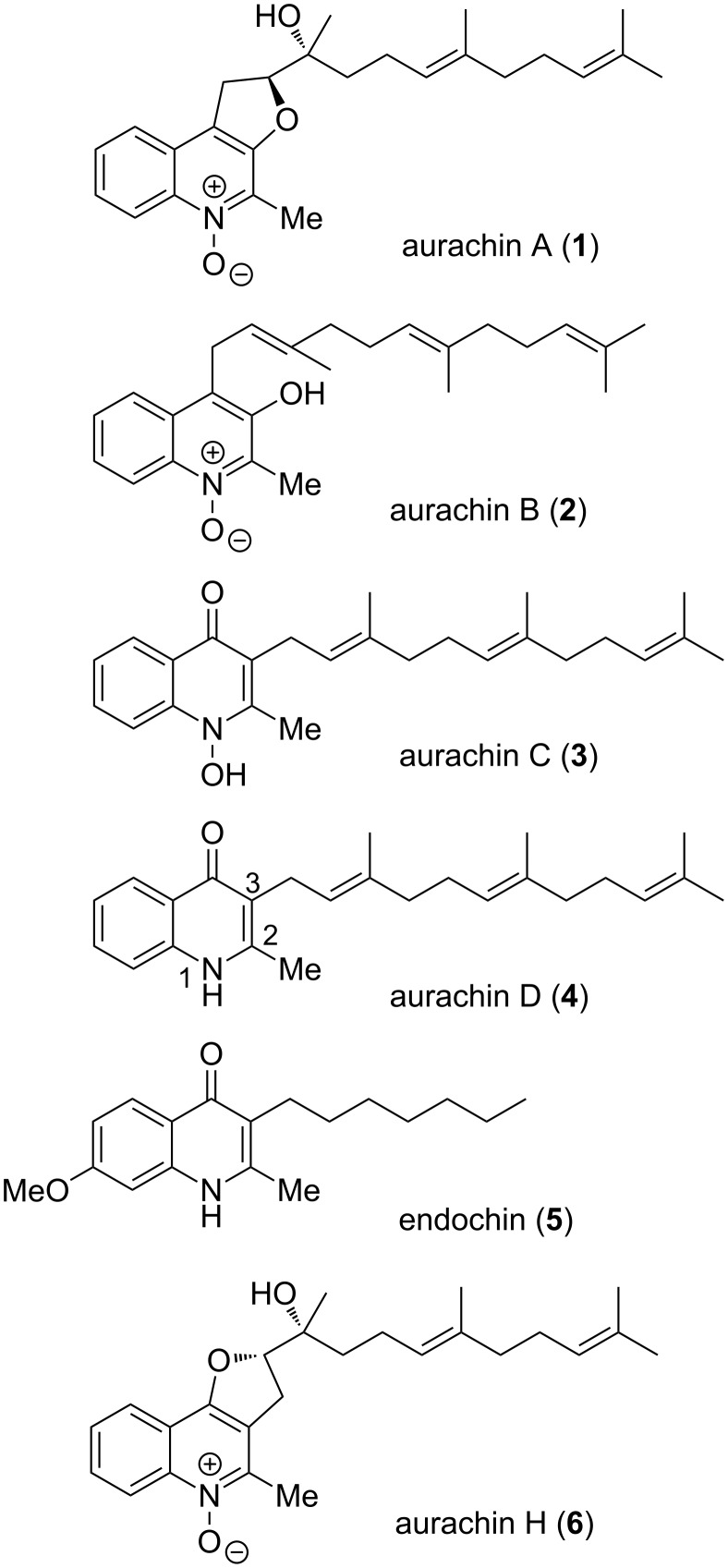
The 2-methyl-4(1*H*)-quinolone compounds: aurachins and endochin.

The four aurachins A–D (**1**–**4**) were first isolated from the myxobacterium *Stigmatella aurantiaca* strain Sg a15 in 1987 (Figure 1) [1]. This class of compounds is characterized by a quinolone or quinoline nucleus substituted in position 3 or 4 by a farnesyl chain. In addition, aurachins can further be functionalized by biosynthetic oxidative processes [2–5]. Since then, additional members of this natural product family were discovered, namely aurachins E–R, from the same strain [3,6], from *Stigmatella erecta* [7] or from *Rhodococcus* sp. Acta 2259 [8].

Initially, aurachins were found to block NADH oxidation in mammalian submitochondrial particles (aurachins C and D being the most active) [1] by the inhibition of complexes I and III of the mitochondrial respiratory chain [9–10]. They are indeed analogues of the physiological ubiquinol and vitamins K. They also affect the photosystem II and the cytochrome *bf* complex of thylakoids in photosynthetic microorganisms [11]. Deeper studies led to the conclusion that these compounds are powerful inhibitors of the quinol oxidation sites of bacterial cytochromes *bo* and *bd*, with dissociation constants in the range of 10 nM. Aurachin D (**4**) was shown to selectively inhibit the cytochrome *bd* complex [12].

With such biological properties, aurachins and their analogues are also strong antimicrobial agents. Aurachins C and D (**3** and **4**) have been described as better inhibitors against Gram-positive bacteria compared to aurachins A and B (**1** and **2**) [1]. All were cytotoxic on L929 mouse fibroblasts with IC_50_ values in the range of 1–3 µg/mL and some of them strongly inhibited *Plasmodium falciparum* at an IC_50_ around 20 ng/mL (i.e., aurachins B, C and E, whereas aurachin D was 100- to 200-fold less active) [6]. This antimalarial activity was comparable to that of endochin (**5**) [13–14] and 2-methyl-4(1*H*)-quinolone analogues as determined in the course of important structure–activity optimization work [15]. In addition, many *N-*hydroxyquinolone derivatives with variable side chains displayed strong antiplasmodial activity at IC_50_ of ca. 1 nM [16].

In fact, owing to their occurrence in many natural products [17–18] and to the high frequency of their biological activities, compounds containing the 4(1*H*)-quinolone nucleus may be considered as important privileged structures [19–21] for medicinal purposes. This motivated the work therein, dealing with the first synthesis of aurachin D (**4**). On this occasion, the preliminary synthesis of ring versus chain analogues was undertaken, focusing on variations of the chain length or on the modulation of electron density of the aromatic ring including surface extension. Oxidative conditions were finally considered en route to *N*-oxide analogues, eventually leading to the heterocyclic core of aurachin H (**6**) (Figure 1).

## Results and Discussion

### Synthesis of aurachin D (**4**) and analogues with chain-length variations

**Scheme 1:**
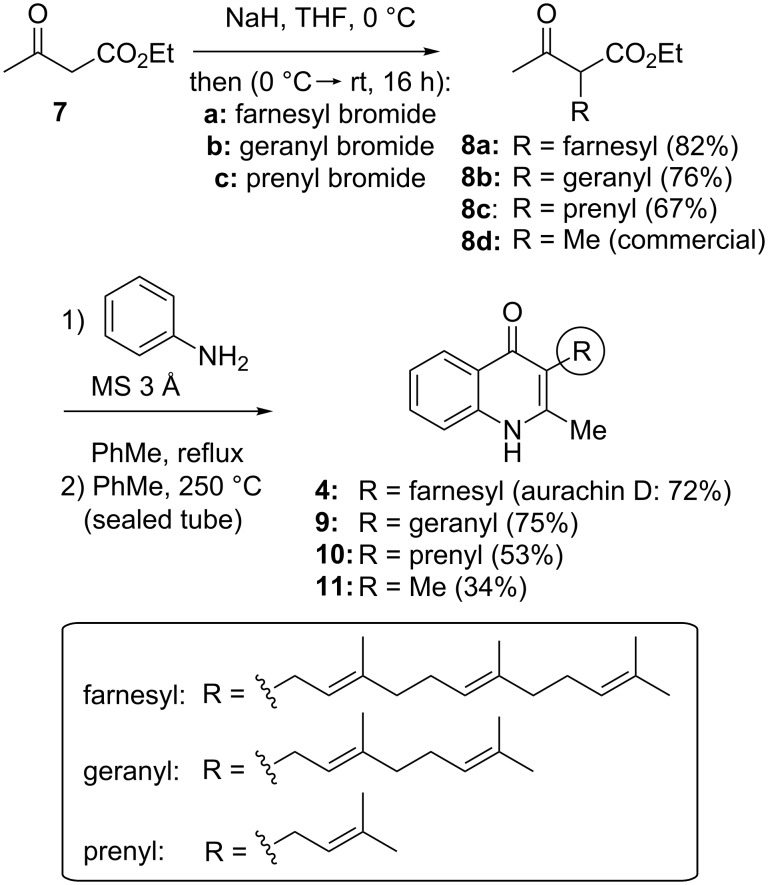
Synthesis of aurachin D (**4**) and geranyl (**9**), prenyl (**10**) and methyl (**11**) analogues.

Aurachin D (**4**) was synthesized in a three-step sequence from ethyl acetoacetate (**7**) (Scheme 1). Alkylation of the sodium salt of **7** in the presence of farnesyl bromide furnished the ethyl 2-farnesyl(acetoacetate) **8a** in 82% yield. Upon treatment with aniline, a Conrad–Limpach process [22–25] gave aurachin D (**4**) in 72% yield. This transformation was performed in toluene under reflux over 3 Å molecular sieves by formation of an imine intermediate, which was cyclized at 250 °C by Claisen condensation. Precipitation of the product by addition of pentane and subsequent filtration gave the pure synthetic aurachin D (**4**) which shared the same spectral properties as the natural product [3]. Analogous compounds bearing a shorter side chain (geranyl: **9** [26], prenyl: **10**, or methyl: **11**) were obtained by the same reaction sequence through the respective 2-alkyl(acetoacetate) **8b–8d**, yet with unexpectedly lower yields for the shorter-chain analogues **10** and **11**.

This method allowed for the rapid gram-scale synthesis of aurachin D (max. 1.1 g made in this work) with good yields and in a minimum of steps, which has to be compared to the extraction yield of the natural product. Indeed from a culture batch of 60 L, the production of compound **4** by *Stigmatella aurantiaca* Sg a15 under basal conditions was described as less than 1 mg/L after about 6 days [1]. This production was improved by the addition of anthranilic acid (i.e., the aurachin biosynthetic precursor) to the culture medium, furnishing 40 mg of **4** from 120 L [3].

### Aurachin D analogues with variations of the aromatic cycles

The same methodology was tentatively applied to other anilines such as 2-naphthylamine (**12**), *p*-anisidine (**13**), 3,4-(methylenedioxy)aniline (**14**), 3,4-difluoroaniline (**15**) and methyl 3-aminobenzoate (**16**), using the 2-farnesyl(acetoacetate) **8a**. Not surprisingly, only the electron-rich anilines **12**–**14** were reactive enough to furnish the expected 3-farnesyl-4(1*H*)-quinolones **17**–**19** in moderate to good yields (Table 1). The electron-poor anilines **15** and **16** were unreactive under these experimental conditions, although the intermediate imine was supposedly formed during the reaction but not isolated.

**Table 1:** Synthesis of aurachin D analogues with aromatic variations.

Starting aniline	Product	Yield

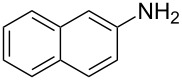 **12**	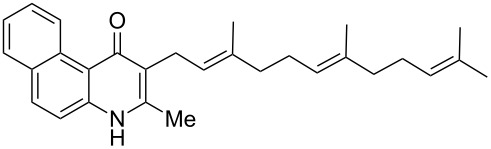 **17**	45%
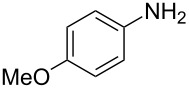 **13**	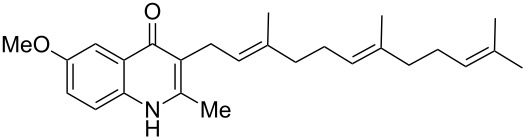 **18**	65%
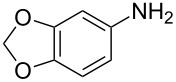 **14**	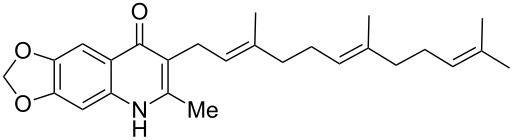 **19**	70%

### The carbocyclic core of aurachin H

**Scheme 2:**
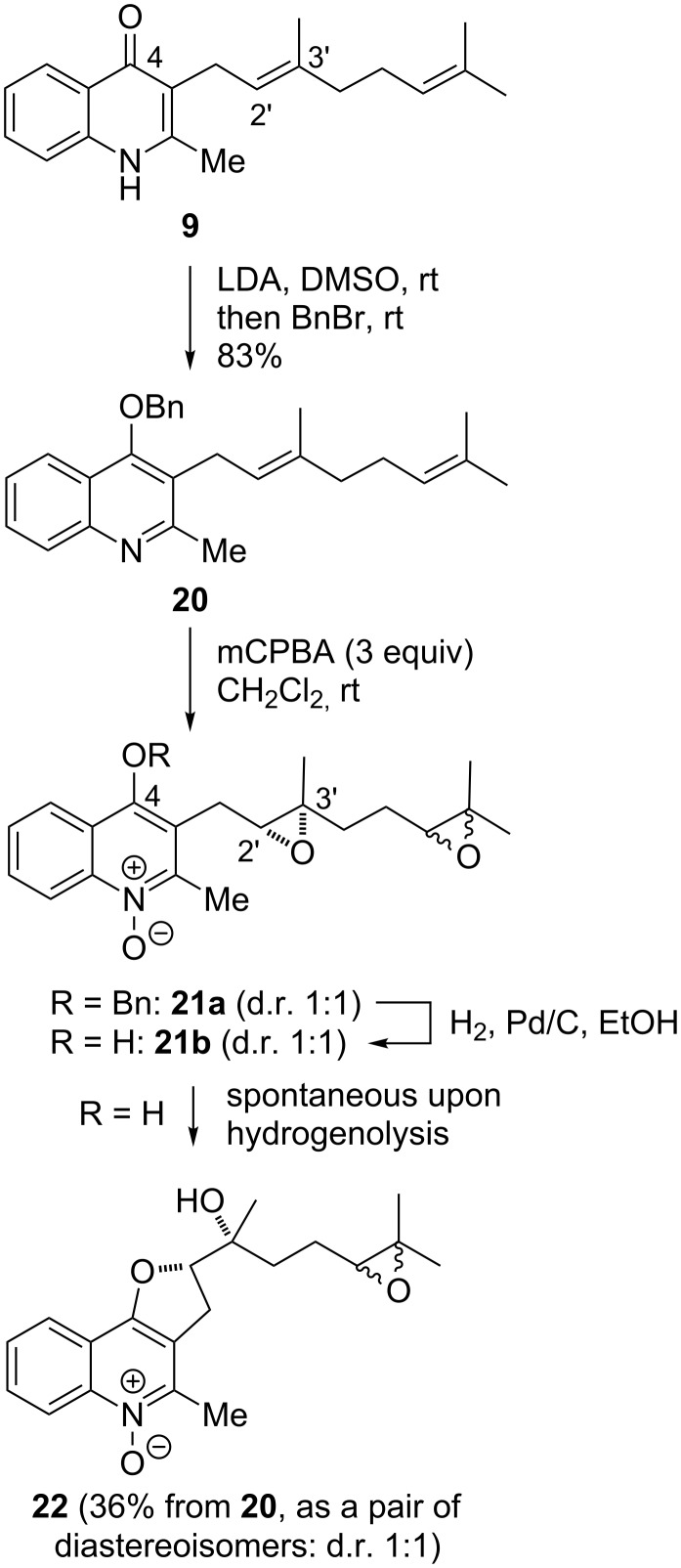
Strategy toward the heterocyclic core of aurachin H.

Aurachin H (**6**) was isolated from *Stigmatella aurantiaca* at the same time as the aurachin series A–L [3]. Biosynthetically, it may arise from the double oxidation of the quinolone nitrogen and the olefin epoxidation (2',3'-position) of the farnesyl chain, with the epoxide being ring-opened with 5-*exo* selectivity by the 4-hydroxy group of the resulting 4-hydroxyquinoline *N*-oxide. Taking the geranyl analogue **9** as a model compound (Scheme 2), after benzylic protection of the 4-hydroxyquinoline form into **20**, oxidative conditions were applied to perform both N-oxidation and olefin epoxidations, in the presence of three equivalents of *m*-chloroperbenzoic acid (mCPBA). The use of smaller quantities of the oxidant only led to complex mixtures of nonselectively oxidized products. The crude product **21a** was then submitted to benzyl group hydrogenolysis. As expected upon completion of the reaction, the 4,5-dihydrofuro[3,2-*c*]quinoline *N*-oxide ring system **22** was obtained as a racemic pair of diastereoisomers in a 1:1 ratio, supposedly formed through the spontaneous cyclization of intermediate **21b**, which was not isolated. We also performed the same reaction sequence from aurachin D (**4**), giving the aurachin-H diepoxide with comparable yields [27]. After optimizing the selectivity of the epoxidation, this reaction may furnish a rapid strategy toward aurachin H (**6**) and related compounds in a bio-inspired manner.

### Biological activities

The cytotoxic activity of aurachin D (**4**) and analogues **9**–**11** and **17**–**19** was evaluated in growth inhibition experiments on mammalian cell lines (Table 2). After 5 days treatment, half inhibitory concentrations (IC_50_) were determined for human HCT-116 colon carcinoma and human K562 myelogenous leukemia cells. The synthesized natural product **4** and its geranyl and methoxy analogues (**9** and **18**) were the most active amongst the tested derivatives with IC_50_ values in the low µg/mL range, whereas analogues **10**, **11**, **17**, and **19** displayed no significant toxicity up to concentrations of 10 µg/mL.

**Table 2:** Cytotoxicity tests on HCT-116 human colon carcinoma and K562 myelogenous leukemia cells (showing IC_50_: half inhibitory concentrations, µg/mL)^a,b^.

Compounds:	**4**	**9**	**10**	**11**	**17**	**18**	**19**

HCT-116	2.23 ± 0.76	2.52 ± 0.48	>10	>10	>10	1.64 ± 0.47	>10
K562	0.65 ± 0.16	1.50 ± 0.31	ca. 10	>10	ca. 10	1.15 ± 0.22	>10

^a^Activities on Vero cells (nontumor cells) were all at IC_50_ >10 µg/mL, with aurachin D (**4**) being cytotoxic at 13.8 µg/mL, except compound **19** at 4.4 µg/mL (the aurachin H analogue **22** was also not active at any concentration up to 50 µg/mL); ^b^All tests performed in triplicate; values were calculated by sigmoidal curve fitting and are displayed as mean ±SD.

**Figure 2:**
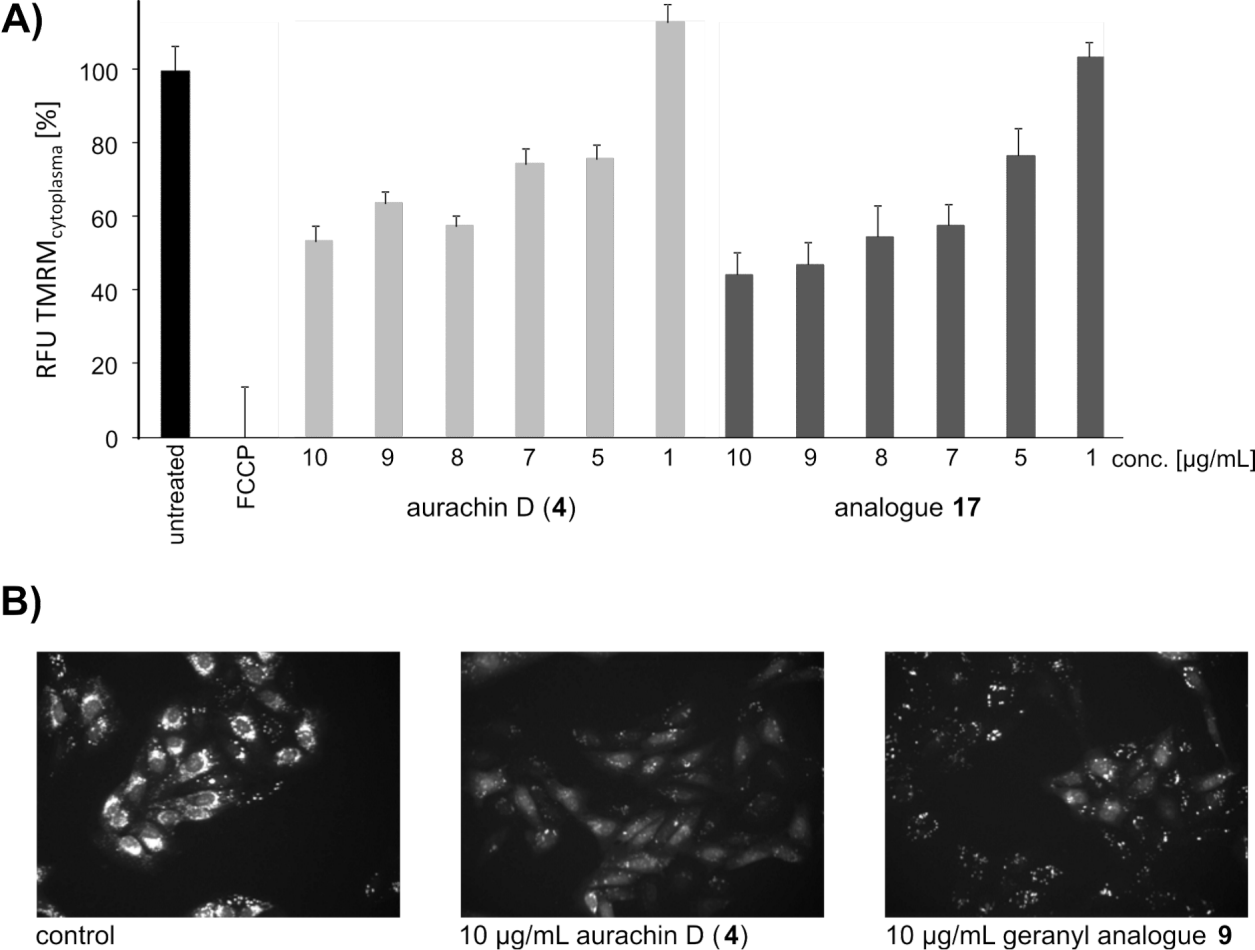
(A) Loss of mitochondrial membrane potential in human U-2 OS osteosarcoma cells that were treated with aurachin D (**4**) and analogue **17**. Images were acquired on a BD Pathway855 automated microscope and subsequently processed in AttoVision v1.6.2. The relative MMP was calculated based on TMRM fluorescence intensity in the cytoplasmic segments. The value of the negative control (untreated cells) was set to 100% and the value of FCCP-treated cells (positive control) was set to 0%. Bars represent the mean ±SEM of all cellular segments within a well. (B) Representative HCS images of U-2 OS cells that were treated with aurachin D (**4**) or the geranyl analogue **9**. Cells were labeled with TMRM and imaged in the rhodamine channel at 200× magnification.

Since aurachins are described as electron-transport inhibitors of the mitochondrial respiratory chain (complex I and III) [28], we analyzed the synthetic analogues in high-content screening experiments with respect to a decrease of the mitochondrial membrane potential (MMP). Human U-2 OS osteosarcoma cells were treated for 16 hours with aurachins **4**, **9**–**11**, and **17**–**19** at concentrations between 1 and 10 µg/mL, and mitochondria were labeled with tetramethylrhodamine methyl ester (TMRM) in order to assess changes in MMP (Figure 2). Most prominent concentration-dependent effects were determined for aurachin D (**4**) and one of its analogues (**17**). At concentrations as low as 5 µg/mL, **4** and **17** significantly reduced the MMP, which was further diminished at higher concentrations (Figure 2A). Other analogues with aromatic variations (**18** and **19**) were essentially inactive in the tested concentration range, although aurachin analogue **18** was determined to have a cytotoxic activity (IC_50_) at ca. 1 µg/mL. This might be due to differential effects on different cell lines, a different time course of electron-transport inhibition, or a different mode of inducing apoptosis (e.g., via enhanced ROS formation) [29]. However, at the highest assay concentration also shorter chain analogues **9**–**11** reduced the MMP to approximately 60–80% of the control value as exemplarily shown for geranyl analogue **9** (Figure 2B).

Antiparasitic activities were evaluated on the chloroquine-resistant *Plasmodium falciparum* FcB1 and on *Trypanosoma brucei gambiense*. Aurachin D (**4**) and its geranyl analogue **9** showed a strong antiplasmodial activity at 0.04 and 0.07 µg/mL, respectively, while analogues **10**, **11** and **17**–**19** were 10- to 100-fold less active (Table 3). Based on the simplest but less active 3-methyl analogue **11**, we compared the activity with the commercial 2-methyl-4(1*H*)-quinolone (**23**), the 3-bromo-2-methyl-4(1*H*)-quinolone (**24**) and *N*-hydroxy-2,3-dimethyl-4(1*H*)-quinolone (**25**), showing the importance of a substituent in position 3. The *N*-hydroxy substitution of **25** had no effect on the activity of the 2,3-dimethylquinolone core (compared to **11**). Due to the lack of the farnesyl chain, this comparison of the quinolone substitution effect yet diverges from previous observations showing an approximately 200-fold higher activity of aurachin C (**3**) compared to aurachin D (**4**) (which however displayed strong activity on our FcB1 strain) [6]. Comparatively, it is known from the literature that farnesol alone is poorly antiplasmodial with an IC_50_ of 64 µM [30], showing the crucial importance of the quinolone core for this strong activity.

**Table 3:** Antiparasitic activities on *Plasmodium falciparum* FcB1 and *Trypanosoma brucei gambiense* (IC_50_, µg/mL).^a^

Compounds:	**4**	**9**	**10**	**11**	**17**	**18**	**19**	**23**	**24**	**25**

*P. falciparum* FcB1	0.04 ± 0.02	0.07 ± 0.02	0.55 ± 0.16	2.30 ± 1.40	0.51 ± 0.18	0.20 ± 0.04	0.42 ± 0.09	7.70 ± 2.50	1.10	2.70 ± 1.30
*T. brucei gambiense*	0.4	1.5	5.8	>10	0.8	1.6	>10	>10	1.7	>10

^a^For *P. falciparum* FcB1, all tests performed in triplicate (except compound **24**); values were calculated by sigmoidal curve fitting and are displayed as mean ±SD.

The antitrypanosomal activity was found much lower than the antiplasmodial activity, with the most active compounds being the natural product **4** and the aromatic analogue **17** at IC_50_ = 0.4 and 0.8 µg/mL, respectively (Table 3). The geranyl (**9**) and prenyl (**10**) analogues still retained some activity at 1.5 and 5.8 µg/mL, respectively. Overall, we found interesting antiparasitic activities for these compounds relative to the cytotoxicity on human Vero cells, with antiplasmodial and antitrypanosomal selectivity indexes of 345 and 35, respectively, for aurachin D (**4**), which still make it medicinally relevant for antiparasitic purposes [31].

In accordance with previous reports [1], antibacterial activities (Table 4) were mainly observed on Gram-positive bacteria while Gram-negative bacteria were not affected, except *Escherichia coli* TolC which is deficient in the corresponding multidrug efflux transporter. The tested compounds were neither active on yeast (*Candida albicans*) nor on molds (*Mucor hiemalis*, *Fusarium oxysporum*, *Alternaria alternata*). The natural compound **4** was one of the most active compounds, but also shorter chain analogues **9** and **10** inhibited, for instance, the growth of *Staphylococcus aureus* at concentrations in the low µg/mL range, whereas analogue **9** was approximately twice as active as **10**. As the 3-methyl analogue **11**, aurachin D analogues with variations of the aromatic cycle (**17**–**19**) were essentially inactive. Solely analogues **18** and **19** displayed a significant growth inhibitory effect on *Bacillus subtilis*.

**Table 4:** Antibacterial activities of 4(1*H*)-quinolone derivatives (MIC_50_, µg/mL).^a^

Compounds:	**4**	**9**	**10**	**11**	**17**	**18**	**19**

Gram-positive							

*Bacillus subtilis* DSM10	<0.1	<0.1	0.22 ± 0.06	49.04 ± 6.26	>64	6.75 ± 0.60	4.66 ± 0.50
*Micrococcus luteus*	1.47 ± 0.12	2.59 ± 0.34	14.31 ± 1.29	>64	>64	49.17 ± 3.94	>64
*Staphylococcus aureus* Newman (MSSA)	5.46 ± 1.05	3.96 ± 0.49	9.66 ± 2.39	>64	>64	>64	34.56 ± 5.13
*Staphylococcus aureus* DSM11822 (MRSA)	2.10 ± 0.19	4.50 ± 0.74	11.3 ± 1.44	>64	>64	>64	>64

Gram-negative							

*Escherichia coli* DH5α (wt)	>64	>64	>64	>64	>64	>64	>64
*Escherichia coli* (TolC)	8.36 ± 1.00	1.41 ± 0.45	6.55 ± 1.36	>64	>64	>64	>64
*Klebsiella pneumoniae* DSM30104	>64	>64	>64	>64	>64	>64	>64

^a^All tests performed in duplicate; values were calculated by sigmoidal curve fitting and are displayed as mean ±SD.

## Conclusion

During this work, not only the total synthesis of aurachin D (**4**) was accomplished, but also that of analogues with variations in chain length or aromatic cycle. The general strategy involved a key Conrad–Limpach synthesis of quinolones. Additionally, we were able to build the carboheterocyclic core of aurachin H (**6**) under epoxidation conditions, i.e., the 4,5-dihydrofuro[2,3-*c*]quinoline *N*-oxide, and thus to synthesize chain analogues of **6**. Biological investigations demonstrated the superiority of the natural product **4** regarding its inhibitory effect on Gram-positive bacteria, human cancer cell lines, and above all on the parasites *Plasmodium falciparum* FcB1 (with an excellent selectivity index of 345) and *Trypanosoma brucei gambiense*. We found that the biological activities were mostly conserved with the geranyl analogue **9**. Also other shorter chain analogues (**10** and **11**) retained some activities. Inhibitory concentrations were, however, by one or two orders of magnitude higher than those determined for aurachin D (**4**) and its geranyl analogue **9**. Variants with modifications of the aromatic cycle (**17**, **18**) were generally less potent although it was found that the methoxy variant **18** is cytotoxic on human cancer cell lines. Indeed, this cytotoxic effect might be rather unspecific or only in part linked to inhibition of the electron transport chain. Finally, the biological activity of the compounds was assessed in a high-content screen on mitochondrial dysfunction. For aurachin D (**4**) and its shorter-chain analogues (**9**–**11**) we found that the mitochondrial membrane potential was reduced to approximately 50% and 60–80% of the control, respectively. Analogues **18** and **19** with variations of the aromatic cycle were inactive up to concentrations of 10 µg/mL. However, analogue **17** strongly diminished the MMP, whereas it remains to be elucidated whether this effect is induced by selectively blocking the electron transport chain or by other apoptotic processes, which, in turn, induce a loss of MMP.

## Supporting Information

Supporting information is available, featuring the synthetic procedures and spectral data for compounds **9**, **10**, **17**–**22**.

File 1 Experimental part.
