# Supplementary material for: Synthesis and biological activities of the respiratory chain inhibitor aurachin D and new ring versus chain analogues
Source: Beilstein J Org Chem. 2013 Jul 31;9:1551–8. doi: 10.3762/bjoc.9.176 (PMC3740496; doi:10.3762/bjoc.9.176)
Supplement: File 1 — Experimental part. [file Beilstein_J_Org_Chem-09-1551-s001.pdf]

Supporting Information

for

Synthesis and biological activities of the respiratory chain inhibitor aurachin D and new ring versus chain analogues

Xu-Wen Li[1], Jennifer Herrmann[2], Yi Zang[1], Philippe Grellier[1], Soizic Prado[1], Rolf Müller*[2] and Bastien Nay*[1]

Address: [1]Muséum National d'Histoire Naturelle, Unité Molécules de Communication et Adaptation des Micro-organismes (UMR 7245 CNRS-MNHN), 57 rue Cuvier (CP 54), 75005 Paris, France and [2]Department of Microbial Natural Products, Helmholtz-Institute for Pharmaceutical Research Saarland (HIPS), Helmholtz Centre for Infection Research (HZI) and Pharmaceutical Biotechnology, Saarland University, Campus C2 3, 66123 Saarbrücken, Germany

Email: Bastien Nay - bnay@mnhn.fr and Rolf Müller - rom@helmholtz-hzi.de

* Corresponding author

Experimental part

General synthetic methods: All reactions were carried out under an atmosphere of argon in distilled anhydrous solvents. THF and toluene were distilled over sodium/benzophenone. CH_2Cl_2 was distilled over CaH_2. Absolute ethanol was used for Pd-catalyzed hydrogenation. Reactions were monitored by TLC on silica gel 60

F_{254}, using UV absorption then vanillin–H_2SO_4 as the staining system. Purifications were performed on silica gel 40–60 µm by flash chromatography. Melting points were measured on a Büchi B-545 apparatus. UV–vis spectra were recorded on a Seconam Uvikon 930 spectrometer. IR spectra were recorded on a Shimadzu 8400S FT spectrometer. NMR spectra were recorded on a Bruker AC 300 spectrometer (Dual probe 5 mm) or on a Bruker Avance I DPX 400 (BBI 1H-BB prode 5 mm). Chemical shifts are given in parts per million and calibrated from residual solvent traces as the internal standard ($CHCl_3$: 7.27 ppm; CHD_2OD: 3.31 ppm; DMSO-d_6: 2.49 ppm). Mass spectra (m/z) were recorded on API QSTAR Pulsar I spectrometer (ESI^+).
Compounds **4** [1], **8a** and **8b** [2], **8c** [3] and **11** [4] displayed similar data to those reported in the literature.

General synthesis of the 3-alkyl-2-methyl-4(1*H*)-quinolones: A 0.1 M solution of the 2-alkyl(acetoacetate) (**8a**–**8d**, 1 equiv) in toluene was heated under reflux for 16 hours in the presence of the aniline (1.1 equiv) and 1 g of dried molecular sieves 4 Å per 0.1 g of the β-ketoester (molecular sieves 3 Å can also be used). After cooling, filtration of the reaction mixture over Celite and evaporation, the residue was diluted in toluene again (0.1 M solution) and heated at 250 °C in a sealed tube for 3 hours. After cooling, unless a clear precipitate was obtained and then directly filtrated (as in the case of **19**), the solution was evaporated, then diluted in a minimum of ether before adding hexanes (10 mL/mmol) to precipitate the product. Washing of the solid product with hexanes and drying under vacuum furnished the pure quinolone product (see Scheme 1 and Table 1 for yields).

3-Geranyl-2-methyl-4(1*H*)-quinolone (**9**): Beige powder; mp (uncorrected): 187–189 °C; R_f 0.4 (CH_2Cl_2/MeOH 20:1); ^1H NMR (CD_3OD, 300 MHz) δ 8.21 (d, J =

8.2 Hz, 1H, 5-H), 7.59 (t, *J* = 8.2 Hz, 1H, 7-H), 7.47 (d, *J* = 8.2 Hz, 1H, 8-H), 7.32 (t, *J* = 8.2 Hz, 1H, 6-H), 5.06 (m, 2H, 2'-H, 6'-H), 3.37 (d, *J* = 6.6 Hz, 2H, 1'-H), 2.43 (s, 3H, 2-CH_3), 1.97–2.07 (m, 4H, 4'-H, 5'-H), 1.77 (s, 3H, 8'-H), 1.59, 1.53 (2 s, 3H each, 3'-CH_3, 7'-CH_3) ppm; ^{13}C NMR (CD_3OD, 75 MHz) δ 178.4 (C-4), 149.8 (C-2), 140.5 (C-8a), 136.0 (C-3'), 132.6 (C-7), 132.1 (C-11'), 126.3 (C-10'), 125.4 (C-6), 125.2 (C-4a), 124.5 (C-2'), 123.7 (C-5), 120.7 (C-3), 118.6 (C-8), 40.7 (C-4'), 27.6 (C-5'), 25.8 (C-8'), 24.7 (C-1'), 18.2, 17.7 (2-CH_3, 7'-CH_3), 16.3 (3'-CH_3) ppm; IR (KBr pellets) ν: 3414, 3098, 2967, 2924, 2855, 1640, 1551, 1497, 1478, 1358, 1254, 953, 756, 567 cm^{-1}; HRMS–ESI^+ (*m*/*z*): $[M + H]^+$ calcd for $C_{20}H_{26}NO$, 296.2022, found, 296.2008.

3-Prenyl-2-methyl-4(1*H*)-quinolone (**10**): Beige powder; mp (uncorrected): 250–252 °C; R_f 0.3 (CH_2Cl_2/MeOH 20:1); ^{1}H NMR (CD_3OD, 300 MHz) δ 8.22 (d, *J* = 8.2 Hz, 1H, 5-H), 7.62 (t, *J* = 8.2 Hz, 1H, 7-H), 7.50 (d, *J* = 8.2 Hz, 1H, 8-H), 7.34 (t, *J* = 8.2 Hz, 1H, 6-H), 5.08 (t, *J* = 6.8 Hz, 1H, 2'-H), 3.38 (d, *J* = 6.8 Hz, 2H, 1'-H), 2.46 (s, 3H, 2-CH_3), 1.80 (s, 3H, 4'-H), 1.68 (s, 3H, 3'-CH_3) ppm; ^{13}C NMR (CD_3OD, 75 MHz) δ 178.6 (C-4), 149.8 (C-2), 140.5 (C-8a), 132.6, 132.4 (C-7, C-3'), 126.3 (C-6), 125.2 (C-4'), 124.5, 123.6 (C-5, C-2'), 120.8 (C-3), 118.6 (C-8), 25.8 (C-4'), 24.9 (C-1'), 18.2, 18.0 (2-CH_3, 3'-CH_3) ppm; IR (KBr pellets) ν: 3414, 3098, 2912, 2881, 1640, 1551, 1497, 1478, 1358, 1254, 949, 756, 571 cm^{-1}; HRMS–ESI^+ (*m*/*z*): $[M + H]^+$ calcd for $C_{15}H_{18}NO$, 228.1382, found, 238.1386.

2-Farnesyl-3-methyl-benzo[*f*]quinolin-1(4*H*)-one (**17**): Beige powder; mp (uncorrected): 148–150 °C; R_f 0.15 (C_6H_{12}/AcOEt 3:1); ^{1}H NMR ($CDCl_3$, 300 MHz) δ 12.30 (s, 1H, NH), 10.60 (d, *J* = 8.7 Hz, 1H, 10-H), 7.95–7.77 (m, 3H, 5-H, 6-H, 7-H),

7.63 (t, J = 7.7 Hz, 1H, 9-H), 7.53 (t, J = 7.7 Hz, 1H, 8-H), 5.25–5.15 (m, 1H, 10'-H), 5.08–4.95 (m, 2H, 2'-H, 6'-H), 3.56 (d, J = 5.7 Hz, 2H, 1'-H), 2.52 (s, 3H, 3'-CH_3), 2.10–1.79 (m, 8H, 4'-H, 5'-H, 8'-H, 9'-H), 1.66 (s, 3H, 12'-H), 1.64 (s, 3H, 7'-CH_3), 1.55 (s, 3H, 11'-CH_3), 1.50 (s, 3H, 3'-CH_3) ppm; ^{13}C NMR ($CDCl_3$, 75 MHz) δ 178.8 (C-1), 144.4 (C-3), 140.3 (C-4a), 135.5, 134.9 (C-3', C-7'), 133.0 (C-6, C-9), 131.6, 131.1, 130.2 (C6a, C-10a, C-11'), 128.1 (C-7), 127.0 (C-8), 125.4 (C-10), 124.3, 124.1 (C-6', C-10'), 123.8 (C-10b), 122.4 (C-2'), 118.5 (C-5), 116.8 (C-2), 39.6, 39.5 (C-4', C-8'), 26.7 (C-5', C-9'), 25.6 (C-12'), 24.7 (C-1'), 17.9, 17.6 (3-CH_3, 11'-CH_3), 16.3, 15.9 (3'-CH_3, 7'-CH_3) ppm; IR (KBr pellets) ν: 3422, 3268, 3044, 2920, 2855, 1628, 1586, 1516, 1485, 1427, 1285, 1258, 829, 748, 575 cm^{-1}; HRMS–ESI$^+$ (*m*/*z*): [M + H]$^+$ calcd for $C_{29}H_{36}NO$, 414.2791, found, 414.2785.

6-Methoxy-3-farnesyl-2-methyl-4(1*H*)-quinolone (**18**): Beige powder; mp (uncorrected): 178–180 °C; R_f 0.45 (CH_2Cl_2/MeOH 20:1); ^{1}H NMR ($CDCl_3$, 300 MHz) δ 11.82 (s, 1H, NH), 7.75–7.68 (m, 1H, 5-H), 7.59 (d, J = 8.9 Hz, 1H, 8-H), 7.17–7.08 (m, 1H, 7-H), 5.19–4.99 (m, 3H, 2'-H, 6'-H, 10'-H), 3.73 (s, 3H, OCH_3), 3.46 (d, J = 6.0 Hz, 2H, 1'-H), 2.49 (s, 3H, 2-CH_3), 2.21–1.84 (m, 8H, 4'-H, 5'-H, 8'-H, 9'-H), 1.71 (s, 3H, 12'-H), 1.64 (s, 3H, 7'-CH_3), 1.58 (s, 3H, 11'-CH_3), 1.54 (s, 3H, 3'-CH_3) ppm; ^{13}C NMR ($CDCl_3$, 75 MHz) δ 176.2 (C-4), 156.0 (C-6), 146.7 (C-2), 135.0 (C-8a), 135.4, 134.4 (C-3', C-7'), 131.2 (C-11'), 125.1 (C-4a), 124.4, 124.2 (C-6', C-10'), 123.3 (C-7), 122.5 (C-2'), 119.8 (C-8), 119.0 (C-3), 104.2 (C-5), 55.5 (CH_3O), 39.7 (C-4', C-8'), 26.8 (C-5', C-9'), 25.7 (C-12'), 24.4 (C-1'), 18.5 (2-CH_3), 17.7 (11'-CH_3), 16.4, 16.0 (3'-CH_3 and 7'-CH_3) ppm; IR (KBr pellets) ν: 3449, 3260, 3098, 2967, 2855, 1620, 1593, 1498, 1389, 1288, 1234, 1153, 1034, 810, 579 cm^{-1}; HRMS–ESI$^+$ (*m*/*z*): [M + H]$^+$ calcd. for $C_{26}H_{36}NO_2$, 394.2740, found, 394.2735.

7-Farnesyl-6-methyl-[1,3]dioxolo[4,5-*g*]quinolin-8(5*H*)-one (**19**): Slightly yellow powder; mp (uncorrected): 298–300 °C (dec.); R_f 0.45 (CH_2Cl_2/CH_3OH 20:1); ^1H NMR (DMSO-d_6, 300 MHz) δ 11.28 (s, 1H, NH), 7.33 (s, 1H, 9-H), 6.87 (s, 1H, 4-H), 6.08 (s, 2H, 2-H), 5.06–4.92 (m, 3H, 2'-H, 6'-H, 10'-H), 3.18 (d, *J* = 6.6 Hz, 2H, 1'-H), 2.27 (s, 3H, 6-CH_3), 2.08–1.80 (m, 8H, 4'-H, 5'-H, 8'-H, 9'-H), 1.70 (s, 3-H, 12'-H), 1.59 (s, 3H, 7'-CH_3), 1.51 (s, 3H, 11'-CH_3), 1.49 (s, 3H, 3'-CH_3) ppm; ^{13}C NMR (DMSO-d_6/$CDCl_3$ 1:1, 75 MHz) δ 174.2 (C-8), 150.3 (C-3a), 144.3 (C-6, C-9a), 135.8, 134.0, 133.3 (C-4a, C-3', C-7'), 130.3 (C-11'), 123.9, 123.7 (C-6', C-10'), 122.9 (C-2'), 118.7, 117.3 (C-7, C-8a), 101.5 (C-9), 101.3 (C-2), 95.8 (C-4), 38.9, 38.6 (C-4', C-8'), 26.1, 25.9 (C-5', C-9'), 25.3 (C-12'), 23.2 (C-1'), 17.3, 17.2 (6-CH_3, 11'-CH_3), 15.8, 15.6 (3'-CH_3, 7'-CH_3) ppm; IR (KBr pellets) ν: 3458, 3252, 3121, 2967, 2920, 2855, 1640, 1609, 1505, 1489, 1474, 1412, 1269, 1234, 1115, 1038, 934, 868, 552 cm^{-1}; HRMS–ESI^+ (*m*/*z*): $[M + H]^+$ calcd. for $C_{26}H_{34}NO_3$, 408.2533, found, 408.2530.

4-Benzyloxy-3-geranyl-2-methylquinoline (**20**): To a dry DMSO solution (2 mL) of **9** (60 mg, 0.2 mmol) under argon was added dropwise a commercial 2 M solution of LDA in THF/hexanes (120 µL, 1.2 equiv) at room temperature. After being stirred for 1 hour, benzyl bromide (30 µL, 1.2 equiv) was added dropwise to the resulting solution and the mixture was stirred for another 1 hour, before being quenched by the addition of brine (5 mL). The product was then extracted with Et_2O (10 mL × 3), dried over $MgSO_4$, concentrated and then purified by flash column chromatography (silica gel, cyclohexane/EtOAc 3:1) to afford 65 mg of the title compound as a colorless resin (83% yield). R_f 0.6 (cyclohexane/EtOAc 2:1); ^1H NMR (CD_3OD, 300 MHz) δ 7.95 (m, 2H, 5-H, 8-H), 7.59 (dd, *J* = 8.0, 7.2 Hz, 1H, 7-H), 7.44–7.28 (m, 6H, 6-H, Ph-H), 4.96 (m, 4H, 2'-H, 6'-H, PhC*H*$_2$), 3.42 (d, *J* = 5.9 Hz, 2H, 1'-H), 2.58 (s, 3H, 2-CH_3), 1.95–2.06 (m, 4H, 4'-H, 5'-H), 1.67, 1.53, 1.48 (3 s, 3H each, 8'-H, 3'-CH_3, 7'-

CH_3) ppm; ^{13}C NMR (CD_3OD, 75 MHz) δ 162.1, 161.4 (C-2, C-4), 148.6 (C-8a), 138.0 (C-3', ipso-Ph), 132.3 (C-7'), 130.4, 129.6, 129.4, 129.0, 128.4 (ortho-Ph, meta-Ph, para-Ph, C-7, C-8), 127.2 (C-3), 126.9 (C-6), 125.2 (C-5), 124.0 (C-4a), 123.2 (C-6'), 122.8 (C-2'), 78.1 (Ph*C*H_2), 40.5 (C-4'), 27.4, 26.3 (C-1', C-5'), 25.9 (C-8'), 20.9 (7'-CH_3), 17.8, 16.6 (2-CH_3, 3'-CH_3) ppm.

4-Benzyloxy-3-(3,7-dimethyl-3,4;6,7-diepoxy-octanyl)-2-methylquinoline *N*-oxide (**21a**): To a dry CH_2Cl_2 solution (3 mL) of compound **20** (65 mg, 0.17 mmol) under argon was added mCPBA (86 mg, 3 equiv) at 0 °C. The reaction was stirred at room temperature during 1.5 hours and then quenched with a saturated solution of $Na_2S_2O_3$ (3 mL), neutralized by a saturated solution of $NaHCO_3$ (3 mL) and extracted by EtOAc (10 mL × 2). The organic extract was washed with brine (10 mL × 2), dried over $MgSO_4$ and concentrated under vacuo. The crude residue of **21a** (80 mg as a pair of diastereoisomers, d.r. = 1:1) was used without further purification for the next step. R_f 0.75 (CH_2Cl_2/MeOH 10:1); 1H NMR ($CDCl_3$, 300 MHz) δ 8.81 (pseudo-d, *J* = 8.8 Hz, 1H, 8-H), 8.05 (pseudo-d, *J* = 8.1, 1H, 5-H), 7.76 (pseudo-t, *J* = 7.2 Hz, 1H, 7-H), 7.61 (pseudo-t, *J* = 7.6 Hz, 1H, 6-H), 7.53–7.40 (m, 5H, Ph-H), 5.22–5.00 (m, 2H, C*H_2*Ph), 3.29–3.11 (m, 1H, 2'-H), 2.98–2.82 (m, 2H, 1'-H), 2.82 (s, 3H, 2-CH_3), 2.70–2.62 (m, 1H, 6'-H), 1.78–1.49 (m, 4H, 4'-H, 5'-H), 1.39 (s, 3H, 8'-H), 1.24 (s, 6H, 3'-CH_3, 7'-CH_3) ppm; HRMS–ESI^+ (*m*/*z*): $[M + H]^+$ calcd. for $C_{27}H_{32}NO_4$, 434.2331, found, 434.2333.

5,6-epoxy-6-methyl-2-(4'-methyl-5'-oxido-2',3'-dihydrofuro[2,3-*c*]quinolin-2'-yl)heptan-2-ol (**22**): To an absolute EtOH solution (3 mL) of the crude compound **21a** (80 mg) under argon was added 10 wt % Pd on carbon (8 mg, 10 wt %). The atmosphere was flushed with H_2 and the mixture was stirred at room temperature over 2 hours. The

reaction mixture was then filtrated on Celite and purified by flash column chromatography (silica gel, CH_2Cl_2/MeOH 20:1 → 10:1) to afford 20 mg of the title compound as a colorless resin made of a 1:1 mixture of two epoxide stereoisomers (36% yield for two steps). R_f 0.35 (CH_2Cl_2/MeOH 10:1); ^{1}H NMR ($CDCl_3$, 400 MHz; overlapped signals for both diastereoisomers) δ 8.70 (d, *J* = 8.8 Hz, 1H, 6'-H), 7.89 (t, *J* = 6.6 Hz, 1H, 9'-H), 7.71 (dd, *J* = 8.2, 7.5Hz, 1H, 7'-H), 7.53 (t, *J* = 7.5 Hz, 1H, 8'-H), 4.99 (m, 1H, 2'-H), 3.46, 3.21 (2 m, 1H each, 3'-H), 2.79 (m, 1H, 5-H), 2.63 (s, 3H, 4'-CH_3), 1.97–1.73 (m, 4H, 3-H, 4-H), 1.36, 1.34, 1.32 (3 s, 3H each, 1-H, 7-H, 6-CH_3) ppm; ^{13}C NMR ($CDCl_3$, 75 MHz; some distinct but very close signals for both diastereoisomers) δ 153.4 (C-9'b), 144.2 (C-4'), 140.9 (C-5'a), 130.4 (C-7'), 127.0 (C-8'), 121.9 (C-9'), 120.3 (C-6'), 117.0 (C-3'a), 115.8 (C-9'a), 91.2/90.8 (C-2'), 73.0/72.9 (C-2), 64.5/64.4 (C-5), 34.4/34.3 (C-3), 29.7/29.6 (C-3'), 24.9 (C-7), 22.9/22.8 (C-4), 22.6/22.2 (C-1), 18.7 (4'-CH_3), 16.0 (6-CH_3) ppm; IR (NaCl film) ν: 3325, 2970, 2928, 2874, 1605, 1532, 1466, 1412, 1377, 1323, 1265, 1227, 1180, 1069, 1003, 922, 768 cm^{-1}; HRMS–ESI$^+$ (*m*/*z*): $[M + H]^+$ calcd. for $C_{20}H_{26}NO_4$, 344.1856, found, 344.1869.

Cytotoxicity assays: *HCT-116 and K562 cells:* Human HCT-116 colon carcinoma (ACC581) and K562 myelogenous leukemia (ACC10) cells were obtained from the German Collection of Microorganisms and Cell Cultures (Deutsche Sammlung für Mikroorganismen und Zellkulturen, DSMZ) and were cultured under the conditions recommended by the depositor. For assessing the cytotoxic activities of aurachin analogues, cells from actively growing cultures were harvested and seeded at 6×10^3 cells per well of 96-well plates (CELLBind® surface) in 180 µL complete medium and treated after 2 h equilibration with compounds in serial dilution. Each analogue was tested in duplicate as well as the internal solvent control. After 5 d incubation, 20 µL of 20 mg/mL MTT (thiazolyl blue tetrazolium bromide) in PBS was added and it

was further incubated for 1.5 h [5]. The medium was then discarded and 100 µL 2-propanol/10 N HCl (250:1) was added in order to dissolve formazan granules. The absorbance at 570 nm was measured using a microplate reader (EL808, Bio-Tek Instruments Inc.) and cell viability was expressed as a percentage relative to the respective solvent control. *VERO cells:* VERO cells were seeded into 96-well microplates at 10^5 cells per mL in RPMI supplemented with 10% foetal calf serum. After 24 h, the cells were washed and two microliters of two-fold serial dilutions of each compound (10 mg/mL) was added to 100 µL of medium. Cells were then incubated for 48 h at 37 ° C under a 5% CO_2 atmosphere. At the end of incubation, 20 µL of MTT solution (5 mg/mL) was added to each well and further incubated for 4 h at 37 °C to allow the formation of formazan. The crystals of formazan were then dissolved with 100 µL of a freshly prepared solution of sodium dodecyl sulfate (SDS) 10% (15 mL) and HCl 1 N (150 µL). The optical density of each well was measured at 595 nm using a multiwell plate reader. The 50% inhibition concentration was then determined by curve fitting.

MMP assay: Human U-2 OS osteosarcoma cells (HTB-96) were obtained from the American Type Culture Collection (ATCC) and were cultured under the conditions recommended by the depositor. Cells from actively growing cultures were harvested and seeded at 8 × 10^3 cells per well in 96-well imaging plates (BD Falcon). After 1 d cells reached approximately 60% confluency and were treated with aurachin analogues at the assigned concentrations for 16 h. In addition, cells were stimulated with 1 µM FCCP [carbonyl cyanide 4-(trifluoromethoxy)phenylhydrazone] as a positive control 1 h prior to the measurement. For labeling, cells were washed twice with assay buffer (135 nM NaCl, 5.4 mM KCl, 2 mM $MgCl_2$, 10 mM glucose, 2 mM $CaCl_2$, 10 mM HEPES; pH 7.35) and 100 µL of a staining solution (50 nM TMRM,

5 µg/mL Hoechst33342 in assay buffer) was added. The cells were stained for 30 min at 37 °C and after washing with assay buffer the samples were examined on an automated microscope (BD Pathway855) in 200-fold magnification with appropriate filter sets for rhodamine and Hoechst. For image analysis, a dual mask (Cyto-Nuc-Ring) was applied in AttoVision v1.6.2. Presented rhodamine fluorescence intensity values were generated in the cytoplasmic segment (ring around nuclei) on a single cell basis and averaged over the complete sample (well).

In vitro antiplasmodial assays: The chloroquine-resistant strain FcB1/Colombia of *Plasmodium falciparum* was maintained in vitro on human erythrocytes in RPMI 1640 medium supplemented by 8% (v/v) heat-inactivated human serum, at 37 °C, under an atmosphere of 3% CO_2, 6% O_2, and 91% N_2. In vitro drug susceptibility assays was measured by [^{3}H]-hypoxanthine incorporation as described [6]. Drugs were prepared in DMSO at a 10 mg/mL concentration. Compounds were serially diluted two-fold with 100 µL culture medium in 96-well plates. Asynchronous parasite cultures (100 µL, 1% parasitaemia and 1% final hematocrite) were then added to each well and incubated for 24 h at 37 °C prior to the addition of 0.5 µCi of [^{3}H]-hypoxanthine (GE Healthcare, France, 1 to 5 Ci·mmol/mL) per well. After a further incubation of 24 h, plates were frozen and thawed. Cell lysates were then collected onto glass-fiber filters and counted in a liquid scintillation spectrometer. The growth inhibition for each drug concentration was determined by comparison of the radioactivity incorporated in the treated culture with that in the control culture maintained on the same plate. The concentration causing 50% growth inhibition (IC_{50}) was obtained from the drug concentration–response curve and the results were expressed as the mean values ± standard deviations determined from several

independent experiments. Chloroquine diphosphate (Sigma–Aldrich) was used as antimalarial drug control.

Antitrypanosomal activities: Bloodstream forms of *Trypanosoma brucei gambiense* strain Feo were cultured in HMI9 medium supplemented with 10% FCS at 37 °C under an atmosphere of 5% CO_2. Antitrypanosomal assays were performed as previously described [7]. In all experiments, log-phage cell cultures were harvested by centrifugation at 3,000*g* and immediately used. Drug assays were based on the conversion of a redox-sensitive dye (resazurin) to a fluorescent product by viable cells. Drug stock solutions were prepared in pure DMSO. *T. b. gambiense* bloodstream forms (3×10^4 cells/mL) were cultured in 96-well plates (200 µL per well) either in the absence or in the presence of different concentrations of inhibitors and with a final DMSO concentration that did not exceeded 1%. After a 72 h incubation, resazurin solution was added in each well at the final concentration of 45 µM. Fluorescence was measured at 530 nm excitation and 590 nm emission wavelengths after a further 4 h incubation. Each inhibitor concentration was tested in triplicate. The percentage of inhibition of parasite growth rate was calculated by comparing the fluorescence of parasites maintained in the presence of drug to that in the absence of drug. DMSO was used as a control. IC_{50}s were determined from the dose–response curves with drug concentrations ranging from 100 µM to 50 nM. Pentamidine was used as antitrypanosomal drug control.

MIC determination: Indicator strains were obtained from the German Collection of Microorganisms and Cell Cultures (Deutsche Sammlung für Mikroorganismen und Zellkulturen, DSMZ) or were part of our internal strain collection. All microorganisms were handled under standard conditions recommended by the depositor. Overnight

cultures of bacteria were prepared in EBS medium (0.5% casein peptone, 0.5% proteose peptone, 0.1% meat peptone, 0.1% yeast extract; pH 7.0). Yeasts and molds were grown in Myc medium (1% phytone peptone, 1% glucose, 50 mM HEPES, pH 7.0). The overnight cultures of microorganisms were diluted to OD_{600} 0.01 (bacteria) or 0.05 (yeasts/molds) in the respective growth medium. Serial dilutions of compounds were prepared as duplicates in sterile 96-well plates. The cell suspension was added and microorganisms were grown overnight on a microplate shaker (900 rpm, 30 °C or 37 °C). Growth inhibition was assessed by measuring the OD_{600} on a plate reader (POLARstar, BMG Labtech). MIC_{50} values were determined as averages relative to respective control samples by sigmoidal curve fitting.

References

1. Höfle, G.; Kunze, B. *J. Nat. Prod.* **2008,** *71,* 1843–1849. doi:10.1021/np8003084
2. Li, Z.; Jung, H.; Park, M.; Lah, M. S.; Koo, S. *Adv. Synth. Cat.* **2011**, *353*, 1913–1917. doi:10.1002/adsc.201100215
3. Peña-López, M.; Martínez, M. M.; Sarandeses, L. A.; Pérez Sestelo, J. *J. Org. Chem.* **2010**, *75*, 5337–5339. doi:10.1021/jo100779z
4. Tatsuda, K.; Tamura, T. *J. Antibiot.* **2000**, *53*, 418–421.
5. Mosmann, T. *J. Immunol. Meth.* **1983**, *65*, 55–63. doi:10.1016/0022-1759(83)90303-4
6. Guillon, J.; Moreau, S.; Mouray, E.; Sinou, V.; Forfar, I.; Belisle Fabre, S.; Desplat, V.; Millet, P.; Parzy, D.; Jarry, C.; Grellier, P. *Bioorg. Med. Chem.*, **2008**, *16*, 9133–9144. doi:10.1016/j.bmc.2008.09.038
7. Zelisko, N.; Atamanyuk, D.; Vasylenko, O.; Grellier, P.; Lesyk, R. *Bioorg. Med. Chem. Lett.*, **2012**, *22*, 7071–7074. doi:10.1016/j.bmcl.2012.09.091
